# Genetic evidence for the association between per- and polyfluoroalkyl substances exposure and rheumatoid arthritis risk: A Mendelian randomization study

**DOI:** 10.1097/MD.0000000000050239

**Published:** 2026-08-21

**Authors:** Hang Xu, Jiachen Liu, Xiaorui Qiu, Jiawei He, Weichu Sun

**Affiliations:** a Xiangya Hospital, Central South University, Changsha, Hunan, China; b Department of Pain, The Third Xiangya Hospital and Institute of Pain Medicine, Central South University, Changsha, China; c Health Science Center at San Antonio, University of Texas, San Antonio, TX.

**Keywords:** per- and polyfluoroalkyl substances, perfluorooctanesulfonic acid, perfluorooctanoic acid, rheumatoid arthritis

## Abstract

Per- and polyfluoroalkyl substances (PFAS), particularly perfluorooctanoic acid (PFOA) and perfluorooctanesulfonic acid (PFOS), are persistent environmental pollutants with potential immunotoxic effects. Emerging epidemiological evidence suggests a link between PFAS exposure and autoimmune diseases, including rheumatoid arthritis (RA). However, the causal nature of this relationship remains unclear. Genetic instruments for circulating PFOA and PFOS levels were obtained from genome-wide association studies of European populations, while RA outcome data were sourced from the FinnGen consortium and United Kingdom Biobank. Associations were evaluated using multiple Mendelian randomization (MR) methods to assess the robustness of the findings. The inverse variance weighted method served as the primary approach. Additional methods, including MR-Egger, weighted median, simple mode, weighted mode, maximum likelihood estimation, and robust adjusted profile score, were applied to address potential pleiotropy and weak instrument bias. Sensitivity analyses, including Cochran *Q*-test for heterogeneity, MR-pleiotropy residual sum and outlier (MR-PRESSO) for pleiotropy detection, and leave-one-out analysis, were performed to validate the robustness of the findings. MR analysis identified a significant association between genetically predicted PFOA levels and increased RA risk (odds ratio = 1.87, 95% confidence interval [1.13–3.11], *P* = .015). Similarly, genetically predicted PFOS levels were positively associated with seropositive RA risk (odds ratio = 1.14, 95% confidence interval [1.00–1.29], *P* = .049). Sensitivity analyses confirmed the robustness of these findings, and reverse MR analysis did not indicate bidirectional causality. Our findings provide genetic evidence suggesting a potential association between PFAS exposure and RA risk. Given the widespread environmental persistence of PFAS, further investigation into potential health impacts and strategies to reduce exposure may be warranted. Further mechanistic studies are warranted to elucidate the biological pathways underlying the potential relationship between PFAS exposure and RA.

## 1. Introduction

Per- and polyfluoroalkyl substances (PFAS) are a class of synthetic organic contaminants known for their exceptionally strong fluorine-carbon bonds, which grant them remarkable resistance to degradation. Owing to their water- and oil-repellent properties, these compounds have been extensively used in industrial and consumer applications for over 7 decades.^[1]^ Among the diverse PFAS, perfluorooctanoic acid (PFOA) and perfluorooctanesulfonic acid (PFOS) are the most widely researched and commonly utilized. Human exposure to these chemicals primarily occurs via ingestion, inhalation, and dermal absorption, leading to their accumulation in plasma due to their high binding affinity for serum albumin.^[2]^ Even at minimal concentrations, PFAS have been linked to numerous adverse health outcomes, including metabolic disturbances, immune system impairments, neurotoxicity, reproductive harm, and heightened risks of chronic diseases.^[3]^ Despite stringent regulatory measures in many countries aimed at restricting or banning PFOA and PFOS, their persistence in the environment remains a pressing public health issue.

Rheumatoid arthritis (RA) is a long-term autoimmune disorder marked by chronic synovial inflammation, which progressively leads to joint deterioration and systemic complications. Although significant progress has been made in RA treatment, the condition continues to be a leading cause of disability and diminished quality of life.^[4]^ The development of RA is influenced by multiple factors, including genetic predisposition, environmental triggers, and immune system dysregulation.^[5]^ Recent studies suggest that environmental contaminants, such as PFAS, may play a role in the onset of autoimmune diseases.^[6–8]^ Research has shown that exposure to PFAS can modulate immune activity, promote persistent inflammation, and interfere with hormonal pathways, all of which are linked to autoimmune disease progression.^[9]^ However, despite increasing concerns, the direct causal link between PFOA/PFOS exposure and RA risk remains uncertain. For example, some studies have observed that elevated serum PFOA levels were associated with increased rheumatoid factor, anti-cyclic citrullinated peptide antibody levels, and Disease Activity Score 28, indicating a possible link between PFAS exposure and immune system dysregulation in RA.^[7,10]^ Specifically, this study reported that PFOA concentrations were significantly associated with an increased odds ratio (OR) for RA (OR = 1.998, 95% confidence interval [CI]: 1.623–2.361, *P* = .01).^[8]^ Additionally, Zhao et al identified significant correlations between multiple PFAS and alterations in specific immune markers, further suggesting that PFAS exposure may elevate RA risk in adults. The OR for PFOA was estimated at 2.2 (95% CI: 1.2–3.9), while perfluoroheptanesulfonic acid was linked to an OR of 1.8 (95% CI: 1.0–3.2)^[7]^ On the other hand, some studies have not found a consistent relationship between PFAS exposure and RA incidence, reflecting inconsistencies in the literature.^[6]^ For instance, an National Health and Nutrition Examination Survey study by Qiao et al found no significant association between serum PFAS levels and RA development, instead suggesting that increased exposure to PFAS mixtures was linked to a reduced risk of RA in females (OR = 0.76, 95% CI: 0.62–0.92).^[6]^ These conflicting findings underscore the complexities of establishing a causal relationship in observational studies, which may be influenced by residual confounding and reverse causation. Given these challenges, further research is essential to clarify the potential causal role of PFAS exposure in RA pathogenesis.

In this study, we conducted a bidirectional 2-sample Mendelian randomization (MR) analysis to explore the potential causal link between people's exposure to PFOA and PFOS and the risk of RA. Our hypothesis suggests that heightened exposure to these PFAS may increase RA susceptibility by disrupting immune regulation and promoting inflammation. By establishing a clearer causal connection between PFAS exposure and RA, our findings could offer valuable insights into the environmental factors influencing autoimmune diseases and help shape public health strategies to reduce PFAS-related health risks.

## 2. Methods

### 2.1. Study design

To enhance the reliability and accuracy of causal inferences, this study strictly followed the 3 fundamental assumptions of MR. First, the instrumental variables (IVs) selected must exhibit a strong correlation with the exposure of interest. Second, these IVs should remain independent of any confounding factors that could influence both the exposure and the outcome. Third, the IVs should affect the outcome solely through the exposure, without any alternative causal pathways.^[11]^

If these assumptions are violated, the MR estimates may be compromised. For example, if genetic variants are not randomly distributed concerning the exposure, the observed associations could be driven by confounding factors rather than a genuine causal link. Similarly, if IVs directly influence the outcome independent of the exposure, the estimated causal effect may be misleading. A visual summary of the study design is provided in the Graphical Abstract.

### 2.2. Data source

This study utilized publicly available genetic data from large-scale genome-wide association studies (GWAS) to examine exposure variables. Specifically, IVs for PFOA and PFOS were derived from a genome-wide association analysis of 1091 blood metabolites and 309 metabolite ratios in a cohort of 8299 European individuals from a Canadian population-based study.^[12]^ This dataset includes genetic variants linked to circulating levels of PFOA and PFOS, identified using high-resolution metabolomics and genome-wide genotyping.

The FinnGen consortium (2021 release)^[13]^ is a large-scale genetic research initiative that combines genotype data from Finnish biobank participants with national health registry records. The datasets analyzed in this study encompass various RA-related phenotypes classified under distinct criteria. All participants were of European ancestry, with genetic data aligned to the human genome version 19/genome reference consortium human build 37 genome build. Sample sizes varied across datasets, with RA cases ranging from 1297 to 6329 and corresponding controls between 147,221 and 215,389, depending on the RA subtype (Table S1, Supplemental Digital Content 1)

The United Kingdom Biobank is a large-scale prospective cohort study comprising approximately 500,000 participants, collecting both genetic and phenotypic data across multiple disease conditions. RA-related data were sourced from the Neale Lab (2017) and Medical Research Council Integrative Epidemiology Unit (2018) GWAS datasets, including both self-reported and hospital-confirmed RA cases (Table S1, Supplemental Digital Content 1). All outcome datasets used in this study are publicly available and underwent rigorous quality control to ensure the reliability of genetic analyses. The large sample sizes and population homogeneity enhance statistical power while minimizing biases related to population stratification.

### 2.3. IV selection

Given the constrained sample sizes in the GWAS datasets for PFOA and PFOS, no single nucleotide polymorphisms (SNPs) achieved the standard genome-wide significance threshold of *P* < 5 × 10^−8^. To improve statistical power and minimize the risk of overlooking true associations, we adopted a more lenient threshold (*P* < 1 × 10^−5^) for selecting IVs, aligning with methodologies applied in prior studies facing similar limitations.^[14,15]^

To address potential linkage disequilibrium effects, SNPs were clumped using an *R*^2^ threshold of 0.001 and a clumping window of 10,000 kb, ensuring that only independent variants were retained. Furthermore, SNPs with *F*-statistics below 10 were excluded to mitigate weak instrument bias, applying the following formula:


$$F=\frac{R^{2}\times \left(N-1-K\right)}{\left(1-R^{2}\right)\times K},$$

where r^2^ is the proportion of exposure variance explained by each IV, *N* represents the GWAS sample size, and *K* refers to the number of IVs.

Additionally, palindromic SNPs were removed to ensure proper alignment between exposure and outcome strands. We also screened for potential confounders such as age, body mass index, smoking status, socioeconomic factors, and comorbidities (e.g., hypertension, diabetes) by consulting sources like PubMed, Google Scholar, Open GWAS, and the GWAS Catalog. No direct associations between the selected SNPs and these confounders were found, allowing all IVs to be retained for further analysis.

### 2.4. MR analysis

To estimate the causal relationship between PFOA and PFOS exposure and RA, we used a variety of MR methods to ensure accurate and unbiased inference. The primary method was inverse variance weighted (IVW), which combines individual SNP effects in a meta-analytic framework. The standard IVW assumes that all IVs influence the outcome only through the exposure, without horizontal pleiotropy. To address heterogeneity, we applied both IVW fixed effects (assuming homogeneous IV effects) and IVW multiplicative random effects (which account for varying effect sizes across instruments, offering better robustness to heterogeneity).^[16]^

To further explore pleiotropy, we conducted MR-Egger regression,^[17]^ which detects and corrects for directional pleiotropy by estimating an intercept term that reflects any systematic bias. The weighted median estimator was included to provide reliable causal estimates, even if up to 50% of IVs are invalid. Maximum likelihood estimation was also used to improve efficiency, especially in the presence of moderately weak instruments. Additionally, we employed robust adjusted profile score, which corrects for weak instrument bias and horizontal pleiotropy, enhancing causal effect estimation when SNPs show heterogeneous effects.^[18]^

We also used simple mode and weighted mode estimators, which classify SNPs based on their clustering in effect estimates. The simple mode assumes that the largest cluster of IVs represents valid instruments, while the weighted mode gives more weight to precise SNPs, reducing the impact of outliers. These mode-based approaches offer alternative causal estimates when some IVs might be invalid due to horizontal pleiotropy.

### 2.5. Sensitivity and heterogeneity analyses

To evaluate the robustness of our MR estimates, we conducted several sensitivity analyses. Cochran *Q*-test was used to assess heterogeneity across the selected IVs, as significant heterogeneity may indicate SNP-specific effects that could introduce bias.

To test for horizontal pleiotropy, we applied 2 key methods: the MR-Egger intercept test, which checks if a significant deviation from 0 in the intercept suggests directional pleiotropy, implying that some IVs may affect the outcome through pathways other than the exposure^[17]^; and the MR-pleiotropy residual sum and outlier (MR-PRESSO) test, which detects outlier SNPs that contribute disproportionately to bias and reestimates causal effects after excluding these outliers, ensuring more reliable results.^[19]^

Additionally, a leave-one-out analysis was performed, sequentially removing each SNP to reassess the causal effect. This approach helps determine if any single IV is disproportionately influencing the association. We visualized the results using the “mr leave-one-out plot” function in R to identify influential SNPs.

All MR analyses were conducted using R software (version 4.3.1), utilizing the TwoSampleMR (MRC Integrative Epidemiology Unit, University of Bristol) and MR-PRESSO (version 1.0) packages.^[20]^

## 3. Result

### 3.1. MR analysis

**Figure 1.**
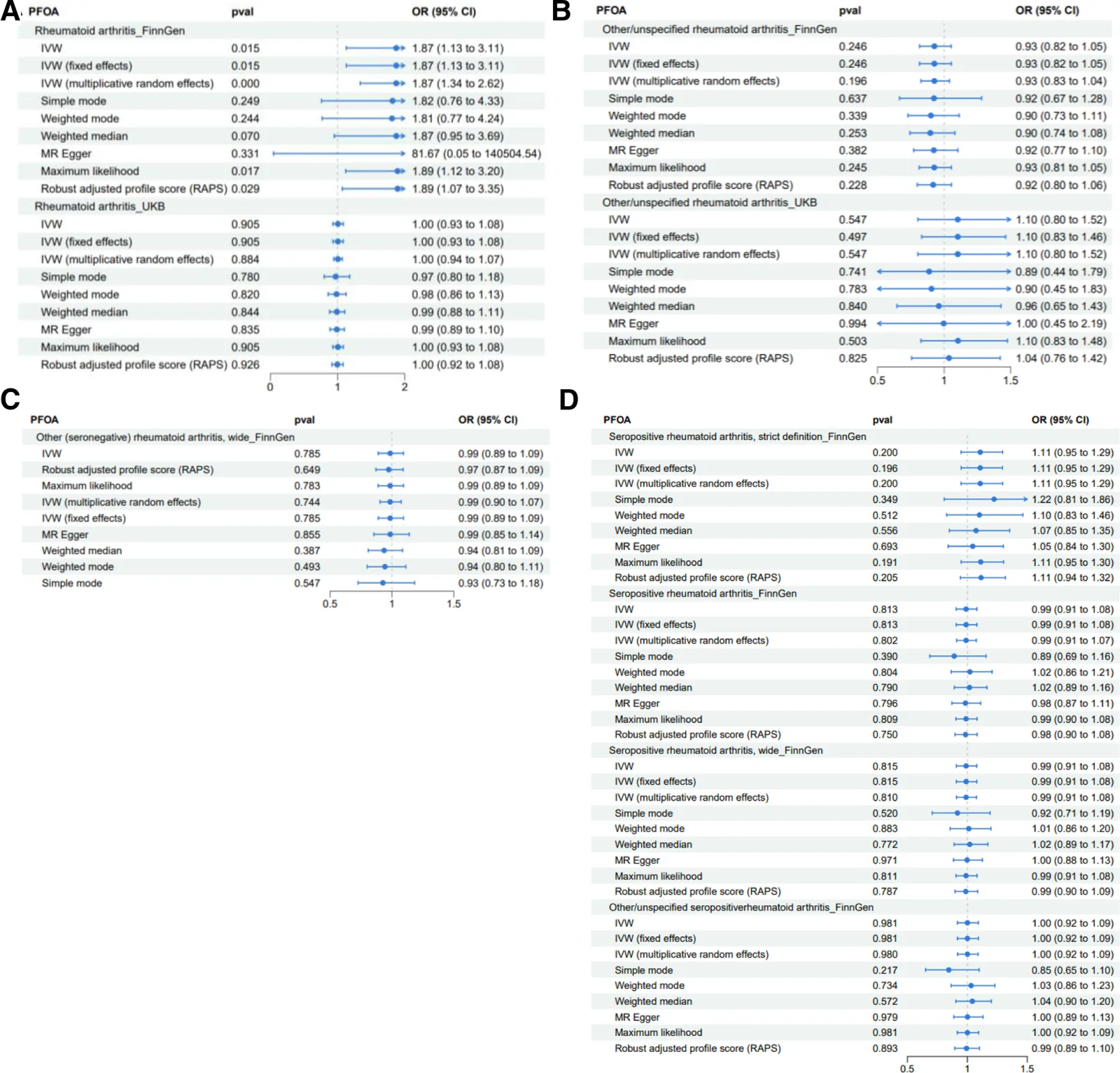
MR estimates for the association between PFOA exposure and RA risk. This figure summarizes the results of multiple MR analyses assessing the causal effect of genetically predicted PFOA levels on the risk of RA. The methods include IVW, MR-Egger regression, weighted median, and weighted mode approaches. Each method provides a point estimate (OR) with corresponding 95% CIs. The vertical line at OR = 1 indicates the null hypothesis (no effect). The consistency of the estimates across methods supports the robustness of the causal inference. SNPs used as IVs were selected based on genome-wide significance and independence (LD threshold *R*^2^ < 0.01). No significant pleiotropy was detected. CI = confidence interval, IV = instrumental variable, IVW = inverse variance weighted, LD = linkage disequilibrium, MR = Mendelian randomization, OR = odds ratio, PFOA = perfluorooctanoic acid, RA = rheumatoid arthritis, SNP = single nucleotide polymorphism, UKB = United Kingdom Biobank.

**Figure 2.**
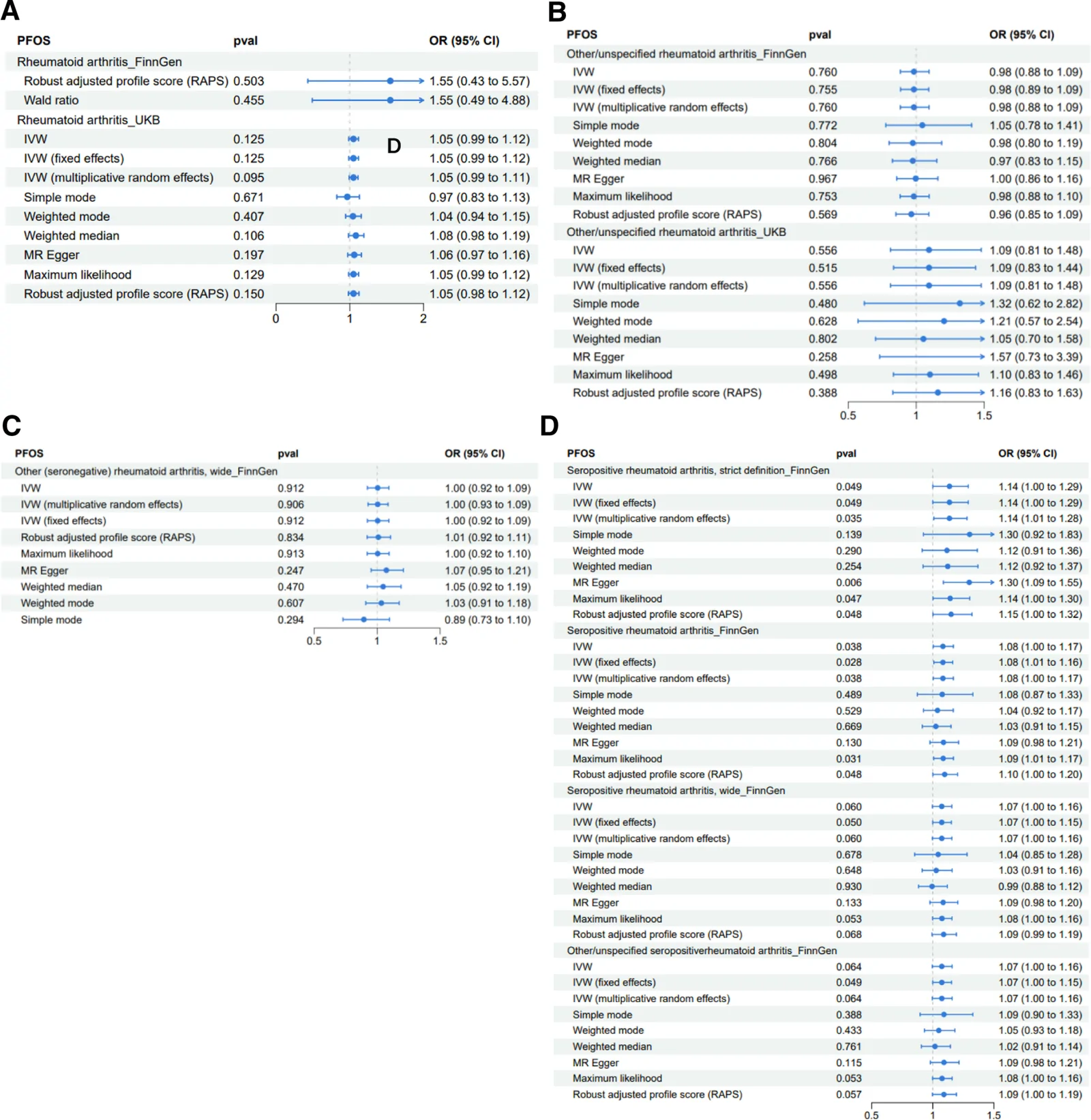
MR estimates for the association between PFOS exposure and RA risk. This figure displays the MR results evaluating the causal association between genetically predicted PFOS levels and RA. Similar to Figure 1. the MR methods used include IVW, MR-Egger, weighted median, and weighted mode. The ORs and 95% CIs are plotted for each method. The figure demonstrates consistency across multiple MR approaches, suggesting a potential causal relationship. IVs were selected based on genome-wide significance and clumping for LD. Horizontal pleiotropy and heterogeneity were tested and found to be minimal, supporting the validity of the estimates. CI = confidence interval, IV = instrumental variable, IVW = inverse variance weighted, LD = linkage disequilibrium, MR = Mendelian randomization, OR = odds ratio, PFOS = perfluorooctane sulfonate, RA = rheumatoid arthritis, SNP = single nucleotide polymorphism, UKB = United Kingdom Biobank.

Our MR analysis revealed a significant causal link between PFOA exposure and an increased risk of RA (OR = 1.87, 95% CI: 1.13–3.11, *P* = .015). Similarly, PFOS exposure was positively correlated with seropositive RA based on the strict definition (OR = 1.14, 95% CI: 1.00–1.29, *P* = .049). Further examination showed that PFOS exposure was also associated with a higher risk of seropositive RA (OR = 1.08, 95% CI: 1.00–1.17, *P* = .038), with a consistent effect observed in the IVW fixed-effects model (OR = 1.08, 95% CI: 1.01–1.16, *P* = .028). Although initial analyses did not show a significant link between PFOS exposure and the broader definition of seropositive RA, statistical significance was reached when the IVW fixed-effects model was applied (OR = 1.07, 95% CI: 1.00–1.15, *P* = .05). A similar trend was observed for RA classified as “other/unspecified seropositive” (OR = 1.07, 95% CI: 1.00–1.15, *P* = .049). (Fig. 1 and 2)

**Figure 3.**
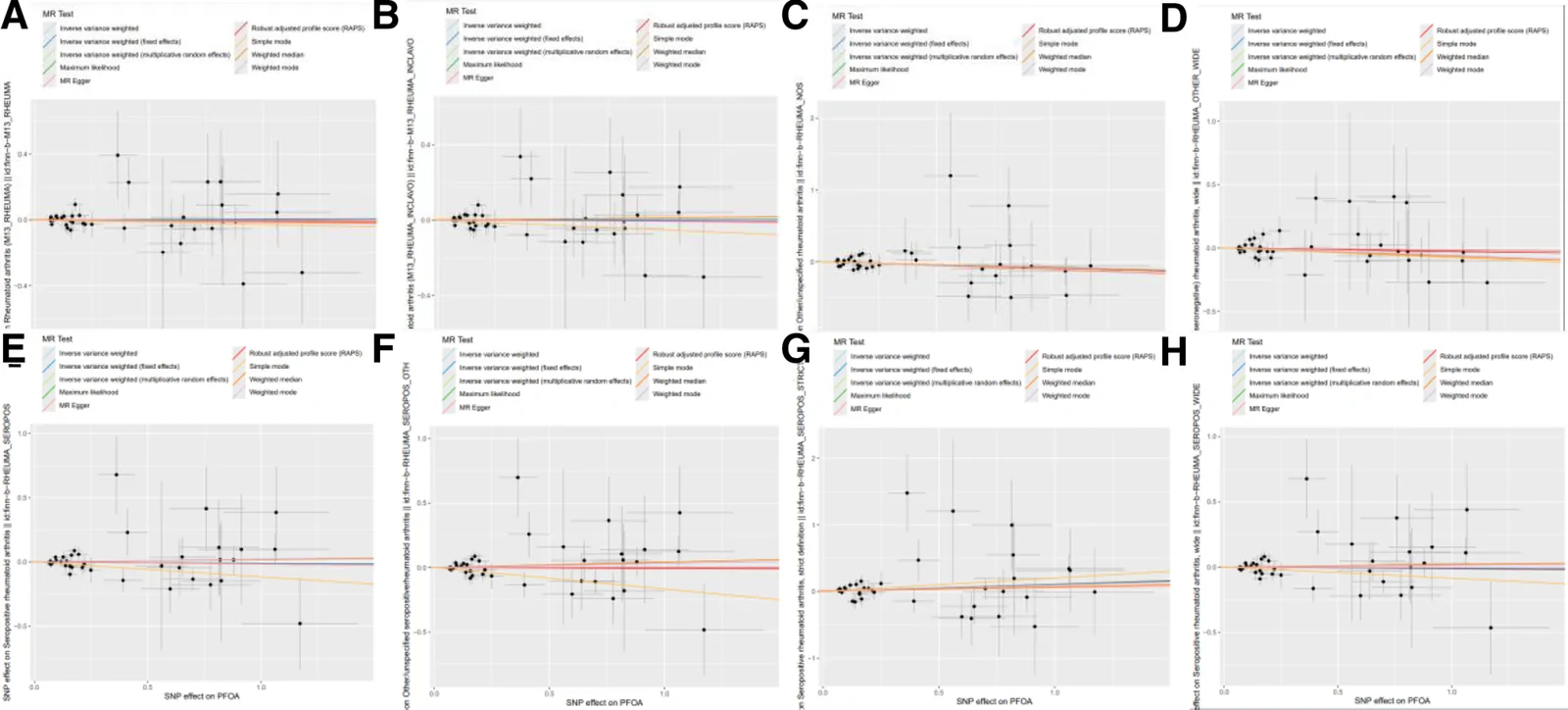
Scatter plots for the causal associations between PFOA and 8 RA phenotypes by 9 MR methods from FinnGen. (A) RA; (B) RA including avohilmo; (C) RA other/unspecified; (D) RA other (seronegative), wide; (E) RA seropositive; (F) RA other/unspecified seropositive; (G) RA seropositive, strict definition; (H) RA seropositive, wide. MR = Mendelian randomization, PFOA = perfluorooctanoic acid, RA = rheumatoid arthritis, SNP = single nucleotide polymorphism.

**Figure 4.**
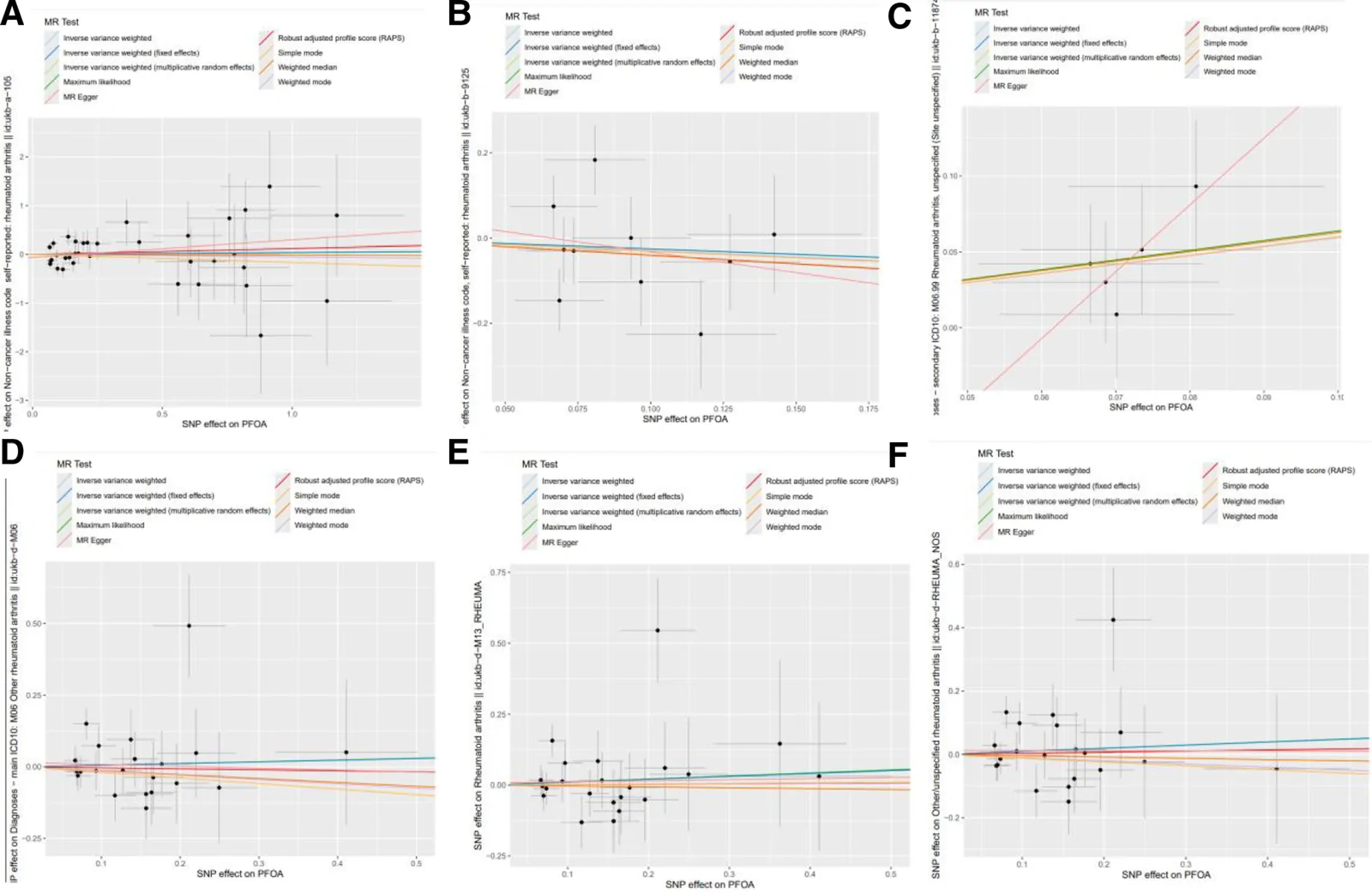
Scatter plots for the causal associations between PFOA and 6 RA phenotypes by 9 MR methods from UKB. (A) RA self-reported (nearlab), (B) RA self-reported (MER_ICU), (C) RA unspecified (MER_ICU), (D) RA other, (E) RA, (F) RA other/unspecified. MR = Mendelian randomization, PFOA = perfluorooctanoic acid, RA = rheumatoid arthritis, SNP = single nucleotide polymorphism, UKB = United Kingdom Biobank.

**Figure 5.**
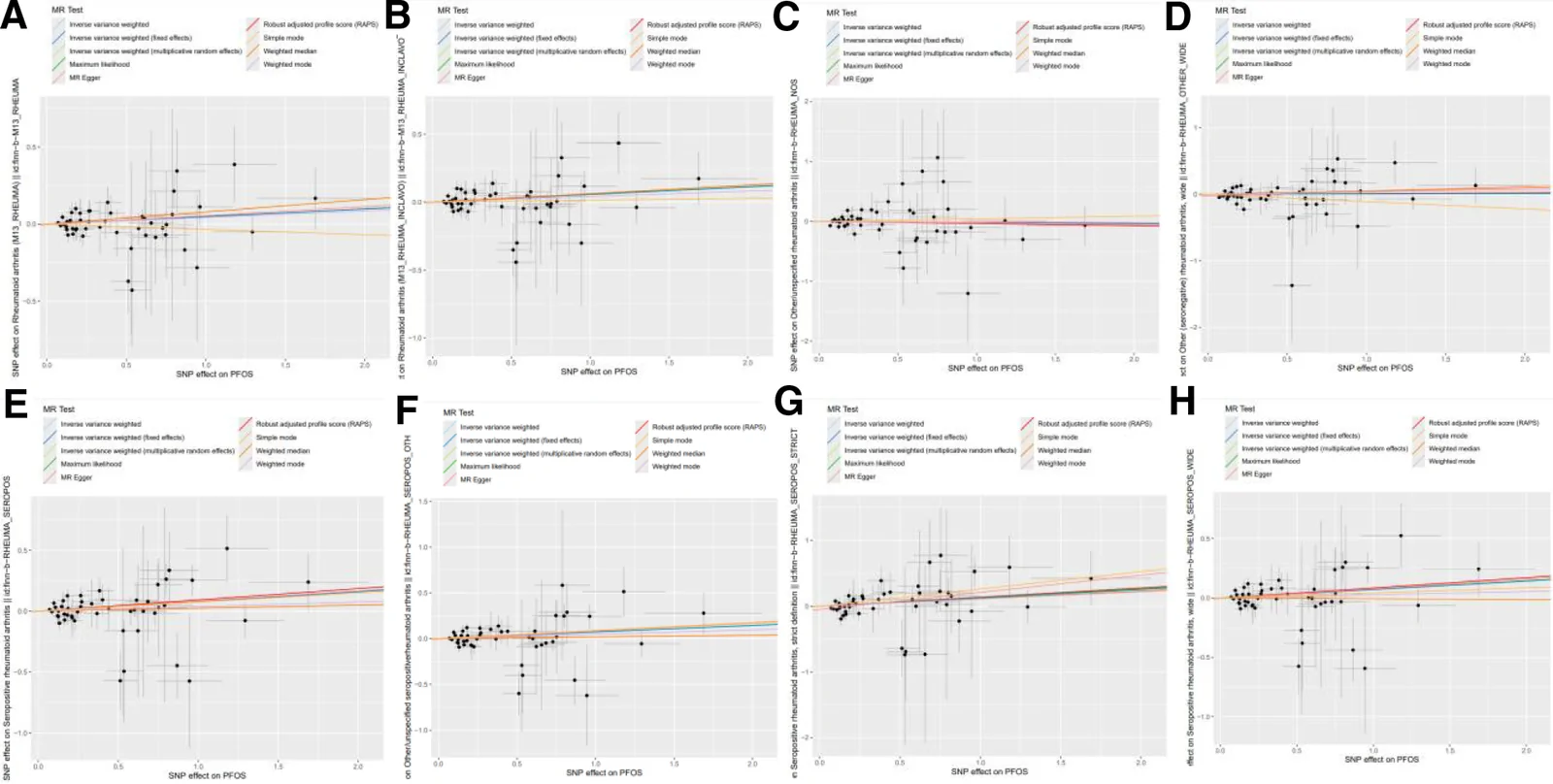
Scatter plots for the causal associations between PFOS and 8 RA phenotypes by 9 MR methods from FinnGen. (A) RA; (B) RA including avohilmo; (C) RA other/unspecified; (D) RA other (seronegative), wide; (E) RA seropositive; (F) RA other/unspecified seropositive; (G) RA seropositive, strict definition; (H) RA seropositive, wide. MR = Mendelian randomization, PFOS = perfluorooctane sulfonate, RA = rheumatoid arthritis, SNP = single nucleotide polymorphism.

**Figure 6.**
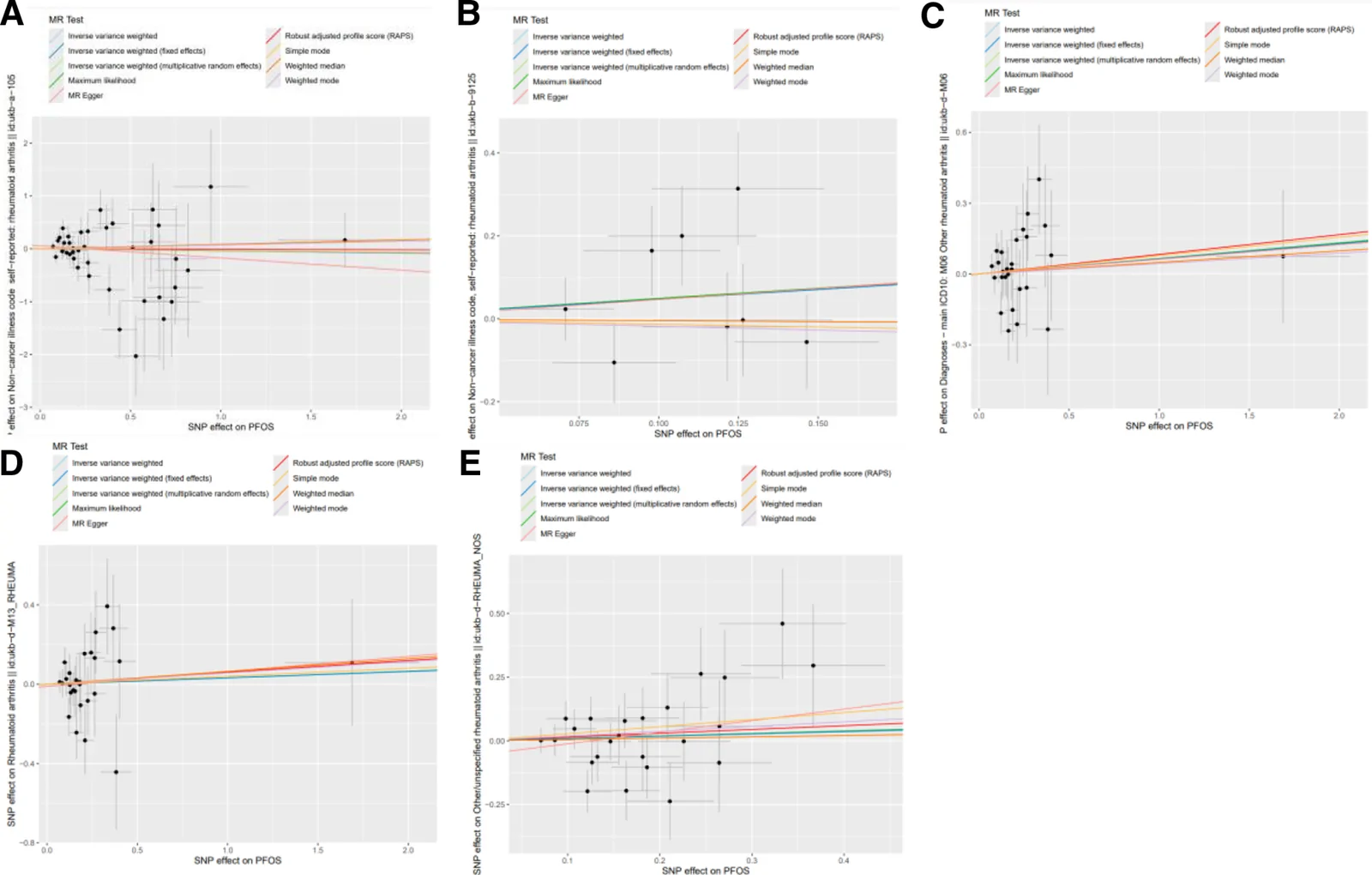
Scatter plots for the causal associations between PFOS and 5 RA phenotypes by 9 MR methods from UKB. (A) RA self-reported (nearlab), (B) RA self-reported (MER_ICU), (C) RA other, (D) RA, (E) RA other/unspecified. MR = Mendelian randomization, PFOS = perfluorooctane sulfonate, RA = rheumatoid arthritis, SNP = single nucleotide polymorphism, UKB = United Kingdom Biobank.

To address potential pleiotropy and heterogeneity, we utilized several complementary MR methods. The weighted median estimator provided results similar to IVW, indicating robustness even if up to 50% of the IVs were invalid. MR-Egger regression revealed no significant directional pleiotropy, as the intercept term was not notably different from 0. Mode-based estimators (simple mode and weighted mode) supported the primary findings, showing a consistent causal link between PFAS exposure and RA across different approaches. The maximum likelihood method and robust adjusted profile score analysis further confirmed these results, reinforcing the statistical reliability of the causal relationship. (Fig. 3–6)

### 3.2. Sensitivity and heterogeneity analyses

To validate the robustness of our findings, we performed several sensitivity analyses. Cochran *Q*-test revealed no significant heterogeneity across the IVs, suggesting that the IVs were statistically consistent (Table S2 and S3, Supplemental Digital Content 2). The MR-PRESSO global test did not detect any outlier SNPs that could have disproportionately influenced the causal effect estimates, further confirming the reliability of our results (Table S4 and S5, Supplemental Digital Content 3).

In the leave-one-out analysis, where each SNP was sequentially excluded, no single SNP was found to significantly impact the overall causal estimates, indicating that our results were not driven by specific variants (Fig. S1, S2, S3, S4, S5, S6, S7 and S8, Supplemental Digital Content 4).

### 3.3. Reverse MR analysis

To eliminate the possibility of reverse causation, we conducted bidirectional MR analyses to examine whether genetically predicted RA affected circulating PFAS levels. The results showed no significant causal effect of RA on PFOA or PFOS concentrations (Table S6, Supplemental Digital Content 5), further supporting the hypothesis that PFAS exposure contributes to RA susceptibility rather than being a result of the disease.

## 4. Discussion

PFAS, commonly known as forever chemicals, are persistent in the environment, accumulate in living organisms, and may pose significant health risks. Chronic exposure to these substances has been linked to immune dysfunction and systemic inflammation, both of which play critical roles in the development of autoimmune diseases such as RA. With increasing global concern over PFAS contamination, several countries have imposed stricter regulations on their use, emphasizing the need for a better understanding of their potential role in autoimmune disease development.^[21]^ This study applies a MR approach to investigate the potential relationship between genetically predicted PFAS exposure and RA risk. Our findings suggest that genetically predicted higher levels of PFOA and PFOS are associated with an increased risk of seropositive RA.

This study provides genetic evidence supporting a potential association between genetically predicted PFOA levels and increased RA risk (OR = 1.87, 95% CI [1.13–3.11], *P* = .015). The mechanisms through which PFAS influence RA are not yet fully understood. We hypothesize that PFAS-induced immune dysfunction may promote the production of pro-inflammatory cytokines, contributing to chronic inflammation and autoimmunity. Previous studies have shown that exposure to 100 μM PFOA increases the secretion of pro-inflammatory cytokines, including tumor necrosis factor-alpha (TNF-α), interleukin (IL)-1, IL-6, and IL-12, all of which are crucial in autoimmune diseases.^[22]^ This effect may be due to an increase in glutathione levels within macrophages after PFOA exposure, which enhances their ability to regulate immune responses through cytokine secretion.^[23,24]^ Since these cytokines are known to contribute to synovial inflammation and joint destruction in RA, PFOA exposure may potentially influence RA susceptibility through inflammatory pathways. These findings raise the possibility that PFOA exposure may influence RA susceptibility through inflammatory pathways.

Our study has several notable strengths. First, the MR approach helps minimize biases from confounding variables and reverse causality, improving the reliability of causal inference. Second, careful SNP selection criteria were applied to improve the reliability of IVs and reduce potential weak instrument bias. Third, multiple sensitivity analyses, including tests for heterogeneity and pleiotropy, supported the robustness of our findings. Additionally, using independent datasets from large-scale GWAS minimized population stratification bias, enhancing the generalizability of our findings.

However, several limitations must be acknowledged. First, our study focused primarily on individuals of European ancestry to reduce population stratification, which may limit the generalizability of our findings to other ethnic groups with different genetic backgrounds and environmental exposures. Second, due to the relatively small GWAS sample sizes for PFOA and PFOS, we used a *P* value threshold of less than 1 × 10^−5^ to select enough SNPs as IVs. While this approach improved statistical power, it may introduce weak instrument bias. Nevertheless, we conducted multiple sensitivity analyses to evaluate the robustness of our results. Third, as with all MR studies, our findings are based on genetic proxies for PFAS exposure, which may not fully reflect real-world exposure dynamics, including bioaccumulation, metabolism, and exposure routes. Residual confounding from unmeasured factors is also a possibility, even though MR helps reduce biases common in observational studies. Additionally, while our focus was on immune-related pathways in RA, we did not examine the broader immunotoxic effects of PFAS, such as their potential impact on other autoimmune or inflammatory diseases. Lastly, while our MR analysis provides genetic evidence supporting a potential relationship between PFAS exposure and RA risk, it does not fully explain the underlying biological mechanisms. Further experimental and mechanistic research, including animal studies, in vitro assays, and dose-response investigations, is needed to clarify how PFAS influence immune dysregulation and RA pathogenesis. Understanding these mechanisms may provide insights into potential preventive strategies and inform future evaluations of PFAS-related health risks.

## 5. Conclusion

Our MR study provides genetic evidence supporting a potential association between genetically predicted PFAS exposure and RA risk. Given the persistent nature of PFAS in the environment, continued evaluation of PFAS-related health risks and strategies to reduce exposure may be warranted. Future research should focus on clarifying the immunopathological mechanisms underlying this association, particularly the role of PFAS-induced immune dysregulation in RA susceptibility. Additionally, studies investigating potential gene–environment interactions and the long-term health effects of chronic PFAS exposure are warranted.

## Author contributions

**Conceptualization:** Hang Xu, Jiachen Liu, Xiaorui Qiu.

**Data curation:** Hang Xu, Jiachen Liu, Xiaorui Qiu, Jiawei He.

**Formal analysis:** Hang Xu, Jiachen Liu, Jiawei He.

**Funding acquisition:** Weichu Sun.

**Writing – original draft:** Hang Xu, Jiachen Liu, Weichu Sun.

**Writing – review & editing:** Hang Xu, Jiachen Liu, Weichu Sun.




























